# A grape seed and bilberry extract reduces blood pressure in individuals at risk of developing type 2 diabetes: the PRECISE study, a double-blind placebo-controlled cross-over intervention study

**DOI:** 10.3389/fnut.2023.1139880

**Published:** 2023-06-07

**Authors:** Teresa Grohmann, Alan W. Walker, Wendy R. Russell, Nigel Hoggard, Xuguang Zhang, Graham Horgan, Baukje de Roos

**Affiliations:** ^1^Rowett Institute, University of Aberdeen, Aberdeen, Scotland, United Kingdom; ^2^By-Health Co., Ltd., Guangzhou, China; ^3^Biomathematics and Statistics Scotland, Aberdeen, United Kingdom

**Keywords:** blood pressure, cardiometabolic health, grape seed extract, bilberry extract, human intervention study, gut microbiota, type 2 diabetes prevention

## Abstract

**Background:**

Type 2 Diabetes Mellitus (T2DM) is a major risk factor for the development of cardiometabolic diseases. T2DM prevention is largely based on weight-loss and whole diet changes, but intervention with dietary plant bioactives may also improve metabolic health.

**Objective:**

To assess whether supplementation with bilberry and grape seed extract for 12 weeks improves cardiometabolic outcomes in individuals at risk of developing T2DM, and to determine whether individual treatment response is associated with differences in gut microbiota composition and levels of phenolic metabolites in blood and feces.

**Methods:**

In the randomized, double-blind, placebo-controlled, cross-over PRECISE intervention study, 14 participants, aged ≥45 years, with a BMI >28 kg/m^2^, and having an increased risk of T2DM, received a supplement containing 250 mg of bilberry plus 300 mg of grape seed extract, or 550 mg of a control extract, per day, for 12 weeks each. Blood samples were obtained for the assessment of HbA1c, fasting glucose, oral glucose tolerance tests, insulin, glucagon levels, total, LDL and HDL cholesterol, and phenolic acids. We also assessed advanced glycation end products in the skin, ambulatory 24 hours blood pressure, 7-day dietary intake by weighed food diaries, fecal levels of phenolic metabolites using LC–MS/MS and gut microbiota composition using 16S rRNA gene sequencing analysis.

**Results:**

The combined bilberry and grape seed extract did not affect glucose and cholesterol outcomes, but it decreased systolic and diastolic ambulatory blood pressure by 4.7 (*p* < 0.001) and 2.3 (*p* = 0.0009) mmHg, respectively. Eight out of fourteen participants were identified as blood pressure ‘responders’. These responders had higher levels of phenylpropionic and phenyllactic acids in their fecal samples, and a higher proportional abundance of *Fusicatenibacter*-related bacteria (*p* < 0.01) in their baseline stool samples.

**Conclusion:**

Long-term supplementation with bilberry and grape seed extract can improve systolic and diastolic blood pressure in individuals at risk of T2DM. Individual responsiveness was correlated with the presence of certain fecal bacterial strains, and an ability to metabolize (epi)catechin into smaller phenolic metabolites.

Clinical trial registry number: Research Registry (number 4084).

## 1. Introduction

Consumption of healthy diets and weight loss are considered effective tools for the prevention and treatment of cardiometabolic diseases, such as type 2 diabetes mellitus (T2DM) (1–4). Current strategies for T2DM prevention and treatment largely focus on weight loss through diet management. Dietary recommendations include consumption of leafy vegetables, fruits, and wholegrains (2, 4, 5). However, weight management by diet can be difficult to maintain, and does not lead to effective weight loss for everyone (6). Therefore, alternative dietary strategies to lower the risk of T2DM, or treat T2DM, are needed (7, 8).

Previous studies show that high consumption of berries and nuts, lowered the risk of T2DM development (4), and consumption of fruit extracts improved glucose and cholesterol metabolism, as well as blood pressure, in individuals with metabolic syndrome and in T2DM patients (9, 10). Furthermore, consumption of phenolic components in fruits, such as anthocyanins and flavan-3-ols, improved markers of cardiometabolic health (11, 12). In particular interventions with berries rich in anthocyanins and flavan-3-ols beneficially affected glucose metabolism in humans (13). Longer-term intervention with bilberry extract, in addition to healthy lifestyle choices, decreased glycated hemoglobin (HbA1c) in T2DM patients (14). Similarly, an acute intervention with bilberry extract reduced postprandial blood glucose after a glucose challenge in T2DM patients (15). *In vitro* experiments and animal studies have mechanistically linked specific phenolic compounds, such as catechin, anthocyanins and epigallocatechin gallate, to the modulation of glucose metabolism (16). For example, epicatechin gallate from green tea reduced uptake of glucose in the small intestine after a glucose challenge, by inhibition of active glucose transport *via* sodium-mediated glucose transporters (SGLT1) across the small intestinal epithelium (16).

The gut microbiota is capable of metabolizing complex flavan-3-ols, which cannot be absorbed in the small intestine, into low molecular weight phenolic acids (12, 17). These phenolic acid metabolites have previously been detected in plasma for prolonged periods, and may be associated with the anti-diabetic effects of fruits and their extracts (11, 12, 18, 19). However, gut microbiota composition and activity can vary greatly between individuals. Therefore, inter-individual differences in gut microbiota composition due to diet, lifestyle factors, use of medication, host genetics, as well as environmental factors, may lead to differences in an individual’s capacity to metabolize dietary phenolic compounds (20), thereby affecting individual responsiveness to intervention with dietary or fruit extracts (21). In addition, BMI may result in different metabolic responses to bioactive compounds of grape extract (22).

The aim of this study was to assess whether a long-term intervention with a supplement containing bilberry and grape seed extract affects glucose and cholesterol metabolism, and blood pressure, in participants at risk of developing T2DM, and thereby reduce their T2DM risk. We also investigated whether factors such as gut microbiota composition and individual bioavailability of phenolic metabolites, such as catechin/epicatechin and phenolic acids, might affect the efficacy of the bilberry and grape seed extract intervention to modulate cardiometabolic outcomes.

## 2. Materials and methods

### 2.1. Recruitment

The PRECISE Study was conducted between May 2018 and September 2020 at the Rowett Institute in Aberdeen, Scotland. Ethical approval for this study was obtained from the Rowett Institute Ethics Committee (2018/ROW_PRECI/1), and the study was registered with Research Registry (number 4084). The study was conducted in accordance with the principles of good clinical practice (GCP) and with the Declaration of Helsinki. All participants provided informed consent prior to starting the intervention study. Study staff complied with the requirements of the Data Protection Act 1998 (until 25th May 2018) and the General Data Protection Regulation (from 25th May 2018) with regards to the collection, storage, processing and disclosure of personal information and upheld the Act’s core principles and adhered to the NHS Scotland Code of Practice on Protecting Patient Confidentiality.

Male and post-menopausal female participants at risk of developing T2DM, or those who were diagnosed with pre-diabetes, were recruited for this study. Eligible participants were aged ≥45 years, had a BMI >28 kg/m^2^, and had HbA1c levels ≥5.5% or a Diabetes Risk Score of 20–24 points [moderate risk, predicting that 1 in 7 people will develop T2DM within 10 years (23–25)]. Exclusion criteria included taking medication affecting glucose metabolism or blood pressure, taking antibiotics, aspirin or aspirin containing drugs, having an allergy or intolerance to the intervention or placebo compounds, diagnosis of diabetes, renal, hepatic or gastro-intestinal disease, or smoking.

Participants were requested to abstain from taking nutritional supplements a month prior to, and during participation in the study. At a screening visit, eligibility was assessed based on HbA1c levels measured in a finger prick whole blood sample (Alere Afinion™ HbA1c Dx analyzer, Afinion™ HbA1c assay, Abbott), measurement of weight, height and waist circumference, and calculation of BMI, to allow the calculation of the Diabetes Risk Score (24). Data on medical history, habitual exercise (validated IPAQ questionnaire), and fruit consumption, were collected through questionnaires.

### 2.2. Study design

The study was designed as a 24-week double-blind, randomized, placebo-controlled, crossover design trial, with each participant receiving the extract (intervention) or placebo (control) treatment for 12 weeks each, in random order, without a wash out period. Participants were randomized in blocks of four into two treatment sequences (control/intervention or intervention/control) via a randomization matrix. The primary outcomes of the study were HbA1c levels, and total, LDL and HDL cholesterol. Secondary outcomes included 24 hours ambulatory blood pressure, an oral glucose tolerance test (OGTT), continuous blood glucose measurements for 14 days, levels of insulin and glucagon, accumulation of advanced glycation end products (AGEs) in skin cells, fecal and plasma levels of phenolic metabolites, and fecal microbial composition.

### 2.3. Intervention and control supplements

Participants were asked to consume 250 mg of bilberry extract (Mirtoselect^®^, Indena, Italy) plus 300 mg of grape seed extract (Enovita^®^, Indena, Italy), or 550 mg of a control supplement (microcrystalline cellulose) per day, each of which were provided as two purple-coated capsules with the intervention and control supplements looking identical. The bilberry extract (Mirtoselect^®^) contained 36% anthocyanins, mainly in the form of cyanidin-3-O-glycosides, whereas the grape seed extract (Enovita^®^) consisted of 5–15% procyanidins in the form of catechins and epicatechins monomers (Supplementary Figure S1). Participants were instructed to take one capsule just before breakfast, and a second capsule just before their evening meal. Compliance was assessed by counting the left-over capsules in the returned containers after each treatment period, subtracting the capsules that were given in excess, and dividing by the total number of capsules that were expected to be taken in a treatment period × 100. Compliance and adverse effects were noted during participant visits.

### 2.4. Study measurements

During the baseline visit, we measured the participants’ height and weight and took a 10 ml fasted blood sample from the antecubital vein by venipuncture. Venous whole blood was aliquoted and stored at −70°C for the measurement of HbA1c, or centrifuged at 4°C, 3,000 *g* for 15 min to obtain plasma, which was also aliquoted and stored at −70°C for the measurement of insulin, glucagon, cholesterol levels, and phenolic metabolites. For one participant, venous sampling was not successful and blood HbA1c and cholesterol levels were measured in a finger prick sample using an auto-analyzer (Alere Afinion™ HbA1c Dx analyzer, Afinion™ HbA1c assay, Abbott; Cholestech LDX™, Abbott). An OGTT was performed by instructing the participants to drink a sugar solution containing 75 g of glucose dissolved in 350 ml filtered tap water, within 15 min. Baseline and post-prandial (2 hour) glucose levels were measured in finger prick blood using the HemoCue^®^ analyzer (Radiometer). Participants completed a 7-day weighed food diary in the week prior to the baseline visit. On the day of the baseline visit, they collected a stool sample using a collection kit (Fecotainer, AT Medical BV), which was processed within 5 hours of collection. Stool samples were immediately processed to a fecal slurry (see below) and stored at −70°C until the extraction of DNA.

During both the 12-week intervention and control periods, HbA1c, total, HDL and LDL cholesterol levels were measured in weeks 8, 9, 10, 11, and 12. Insulin, glucagon, fasting and post-prandial glucose levels (OGTT test) were measured at baseline and after 12 weeks of the intervention and control periods. Continuous blood glucose monitoring (FreeStyle Libre, Abbott) was performed during the last two weeks of both intervention and control periods. The FreeStyle libre glucose sensor was applied on the backside of the upper arm, measuring blood glucose levels every 20 min. The participants were provided with a 24 hours ambulatory blood pressure monitor (CardioVisions, PMS Instruments), pre-set to take an automated measurement every 30 min during daytime and every hour during night-time, in week 8 of both intervention and control periods. Advanced glycation end products (AGE) reader measurements were performed by scanning the forearm in week 8 and 12 of both intervention and control periods using the non-invasive AGE reader (Diagnoptics Technologies B.V.), following manufacturer’s instructions, resulting in an AGE risk score. Seven-day weighed food diaries were obtained in the last week of both intervention and control periods, and on the 7th day of weighed food diary data collection, a stool sample was collected and frozen at −70°C until the extraction of and analysis of phenolic metabolites from fecal waters. The participants’ weight was measured in the last week of both intervention and control periods. Exercise habits were evaluated using the International Physical Activity Questionnaire (IPAQ) in the last week of both intervention and control periods. Participants provided information on the frequency of their fruit consumption, in particular how regularly they consumed strawberries, blueberries, grapes and apples (more than once per week, once per week, once per month, less than once per month, or none) at baseline and at the end of the intervention and control period. We instructed study participants to maintain their habitual diet and exercise routines throughout the study period.

### 2.5. Sample preparation and analysis

HbA1c and total, LDL and HDL cholesterol levels were analyzed using a KONELAB 30 analyzer (Thermo Scientific) according to the manufacturer’s instructions. Plasma levels of insulin and glucagon were analyzed by ELISA (Mercodia and Antibodies online, respectively) according to the manufacturer’s instructions. Fasted blood glucose and insulin measurements were used to calculate insulin resistance (HOMA-IR): HOMA-IR = (glucose [mmol/l] × insulin [mU/ml])/22.5, using the following cut-off points: <1 for optimal insulin sensitivity, >1.9 for early insulin resistance and > 2.9 for significant insulin resistance.

Baseline fecal samples were processed into a fecal slurry within 5 hours, as previously described (26). The fecal slurry aliquots were stored at −70°C until microbial DNA extraction was performed using the FastDNA^TM^ SPIN Kit For Soil (MP Biomedicals). After DNA extraction, and PCR amplification with barcode primers for the V1-V2 region of the 16S rRNA gene [MiSeq-27F (5′-AATGATACGGCGACCACCGAGATCTACACTATGGTAATTCCAGMGTTYGATYMTGGCTCAG-3′) and MiSeq-338R (5′-CAAGCAGAAGACGGCATACGAGAT-barcode-AGTCAGTCAGAAGCTGCCTCCCGTAGGAGT-3′)], samples were further purified with ethanol washes and bead clean-up, and then sequenced using the Illumina MiSeq platform at the in-house facilities at the Center for Genomic Enabled Biology and Medicine (CGEBM), University of Aberdeen, UK, to generate bacterial community profiles. Gut microbiota sequence data were analyzed using the mothur software package (27), following the same sample data processing steps described previously (26), and clustering patterns in Bray-Curtis-based dendrograms visualized using the online software iTOL (28), with subsampling being performed at 23,000 sequences to obtain equal sequence depths between samples for comparisons.

Phenolic metabolites in plasma were analyzed as described by Saha et al. (29), using internal standards described by Neacsu et al. (30). Briefly, plasma samples (200 μl) were mixed 1:1 with phosphate buffer (pH 5) and vortexed. Glucuronidase (20 μl, 10,000 U/ml) and sulfatase (20 μl, 1,000 U/ml) enzymes were added, vortexed and incubated for two hours at 37°C to facilitate the removal of moieties. Dimethylformamide (100 μl) and 50% trichloroacetic acid (20 μl) were added and incubated at room temperature for 10 min. The samples were centrifuged (13,000 rpm, 15 min, −3°C), and supernatants were analyzed *via* LC–MS/MS. Frozen fecal samples were thawed overnight, homogenized in a stomacher for two minutes and centrifuged for two hours at 10°C, 50,000 × g. The supernatant containing the phenolic metabolites was collected and analyzed for phenolic metabolites by LC–MS/MS according to Russell et al. (31).

### 2.6. Analysis of dietary intake

Weighed food dietary records obtained at baseline and in the last week of both intervention and control periods were analyzed using WinDiets software. Basal Metabolic Rate (BMR) was calculated from the baseline characteristics weight, height, age and sex^1^ and used to investigate underreporting in food diaries, identified as the average caloric intake being below the BMR.

### 2.7. Statistical analysis

Power calculation was performed using the software package G-Power (32, 33), based on effect sizes of longer-term dietary interventions on HbA1c and cholesterol outcomes in populations with pre-diabetes, T2DM or hypercholesterolemia (31, 34–40). Reported effect sizes were variable; from a selection of these we determined a mean standardized effect size of 0.6, needing *n* = 23 volunteers to have a power of 80%.

Statistical analysis and visualization of results was done in R version 4.0.2 (41) using the ggplot2 (42), and corrplot (43) packages. Normality of data was tested using Royston tests with the package MVN (44). Data for OGTT, insulin and glucagon, ambulatory blood pressure and pulse were recorded at three time points (baseline, control, intervention) and were analyzed using two-way ANOVA and Tukey post-hoc analysis. HbA1c and cholesterol levels, where five timepoint measurements between treatment and control periods were compared, where analyzed with a linear mixed model, using the lme4 (45) and lmerTest (46) packages, with differences between intervention and baseline. The end value for the first intervention period was taken as the baseline value for the second intervention period. The random effects of the linear model were participants and period (first or second), while the fixed effects were (extract, placebo) treatment (measurement) week, and the interaction between treatment and weeks.

The covariates for OGTT, insulin and glucagon were treatment (baseline, intervention, control) and participants, and covariates for blood pressure and pulse analysis were (24 hours, day or night) treatment (extract, control) and participants. To assess whether individual participants were responders or non-responders to intervention, we established within each individual whether there was a significant difference between the 24 hours blood pressure measurements with a two-way ANOVA, with terms for treatment and time. ANOVA results were presented as F-test values (degrees of freedom) and value of p, according to Field et al. (47). 7-Day food diary entries for each period (baseline, intervention and control) were averaged across the week per participant, and differences in total energy, total fat, saturated fat, total carbohydrates, free sugar, fiber and salt intake between the periods were assessed on the study population level by two-way ANOVA and Tukey post-hoc test, with (baseline, intervention, control) period and participant as factors. Data for habitual fruit consumption, weight changes, exercise and AGE reader outcomes were evaluated by Wilcoxon rank test.

Plasma and fecal metabolite composition were compared in a two-way ANOVA and Tukey post-hoc test with covariate (extract, placebo) treatment for the 60 targeted phenolic metabolites. The association between data for individual fecal metabolites and blood pressure (24 hours average, daytime (7 am-11 pm)/night time (11 pm-7 am) averages - data not shown) were assessed with Spearman correlations, calculated using the corrplot function in R (43). Statistical assessment of microbiota clustering patterns was assessed *via* the Parsimony calculation in mothur (27), which tests for significant differences between groups on shared dendrogram branches. The Parsimony calculation was performed to assess general microbiota clustering patterns independent of study outcomes, and dependent on blood pressure response, followed by an Analysis of molecular variance (AMOVA) calculation in mothur (27), which is a non-parametric analysis of differences of microbiota composition between samples, based on a distance matrix. To test for significant differences in specific taxa within the gut microbiota between blood pressure responders and non-responders, Linear discriminant analysis Effect Size (LEfSe) (48) and Metastats (49) were used in mothur (27). LEfSe employs two statistical tests (Kruskal-Wallis and Wilcoxon) to identify significant differences between microbial taxa (e.g., responders, non-responders), and the Linear discrimination analysis (LDA) score is determined by the proportional abundance of the bacteria in the sample, which would explain the effect size (48). Metastats uses a non-parametric t-test to assess the differences between sample cohorts (49).

## 3. Results

### 3.1. Participant characteristics, side effects, and compliance

**Figure 1:**
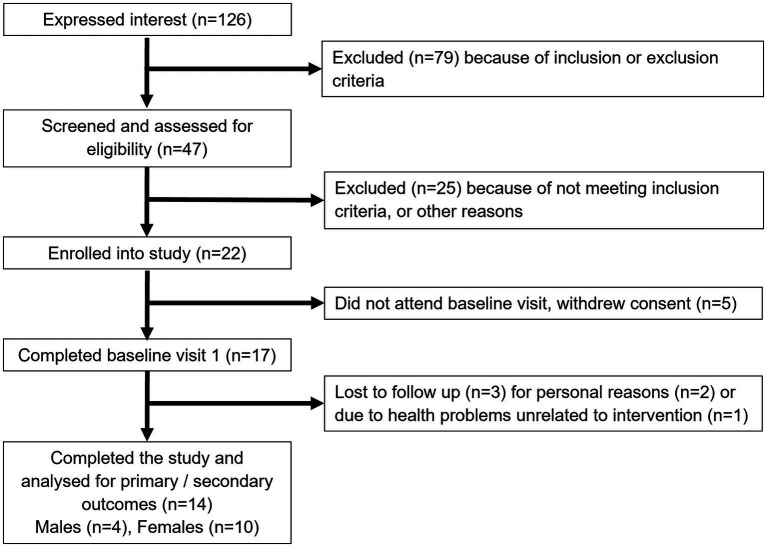
Study flow diagram.

**Figure 2:**
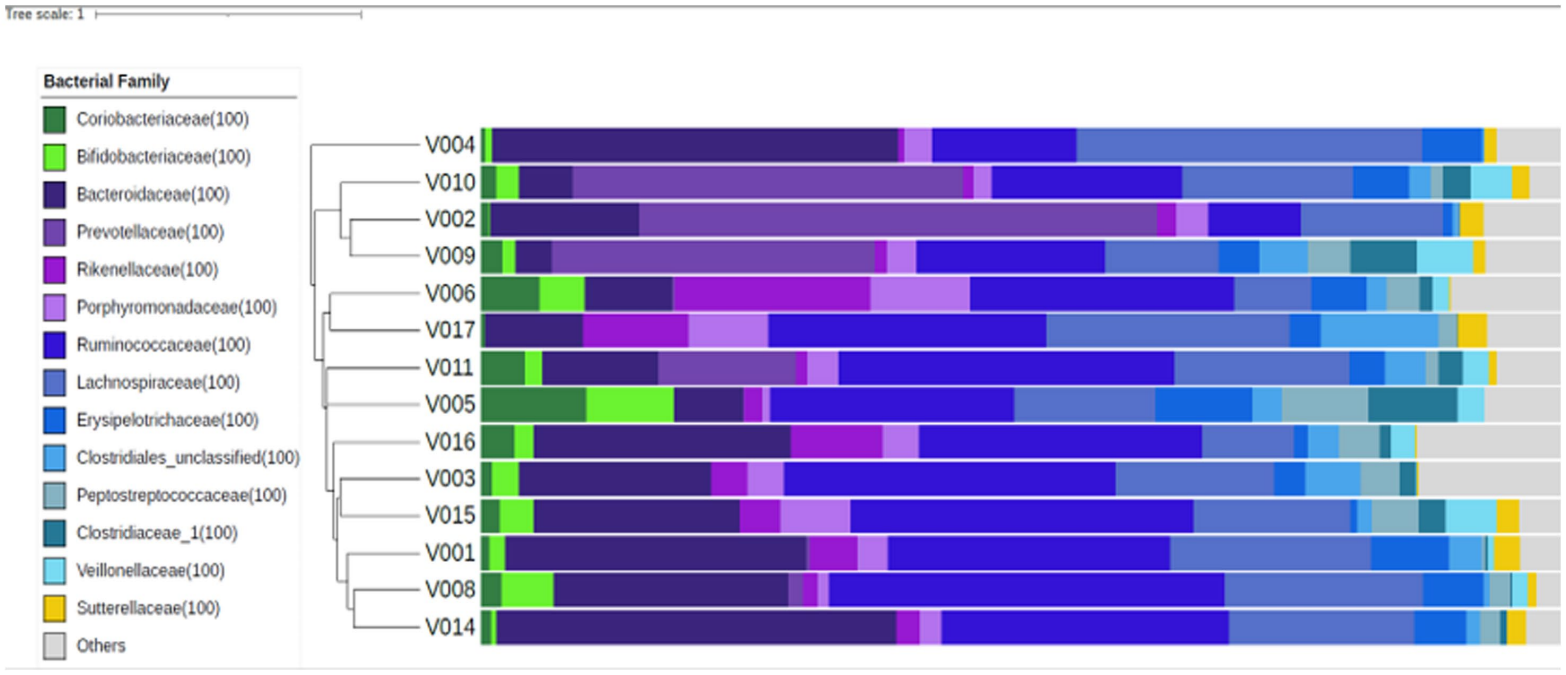
Fecal microbiota composition at baseline, for each study participant (*n* = 14). Similar microbial compositions are grouped together in this Bray-Curtis dendrogram. The proportional abundances of selected bacterial families are presented to the right of the dendrogram and were colored according to the phylum they belong to: *Actinobacteria* – green, *Bacteroidetes* – purple, *Firmicutes* – blue, *Proteobacteria* – yellow, and others – grey.

Figure 1 shows the study flow diagrams. Informed consent to participate in the study was obtained from 22 participants – seven males and 15 females. Five participants withdrew from the study prior to the baseline visit, and three participants terminated participation during the study. Fourteen participants (four males and 10 females), all Caucasian, completed the study. The baseline characteristics of the participants are summarized in Table 1. The diabetes risk was generally determined either by having a family history of diabetes or due to a BMI ≥25 kg/m^2^. Baseline fecal microbiota of participants were mainly comprised of bacteria belonging to the *Firmicutes* and *Bacteroidetes* phyla (Figure 2), which are typically dominant in the colon of most adults (26). One study participant experienced bloating during the intervention period, and one participant reported increased regurgitation during the control period. No other side effects or adverse events were reported during the study. Compliance was 97 ± 6.3% during intervention period and 92 ± 9.3% during the control period. There was no change in the weight or BMI of the participants over the six-month study period (data not shown). Reported energy and salt intakes were statistically higher at the end of the intervention period compared with the control period (*p* = 0.03 and *p* = 0.005, respectively) (Table 2). No differences in the frequency of consumption of fruits (Table 2), or in vigorous exercise bouts (data not shown), were observed between the intervention and control periods.

**Table 1:** Baseline characteristics of participants.

Parameter	Males (n = 4)	Females (n = 10)
Age (yr)	57.8 ± 7.0	57.7 ± 4.7
Body weight (kg)	99.0 ± 11.4	95.2 ± 27.3
BMI (kg/m^2^)	30.3 ± 1.9	38.2 ± 9.6
Waist circumference (cm)	109.8 ± 8.9	111.0 ± 14.4
HbA1c (%)	5.4 ± 0.3	5.4 ± 0.2
Diabetes Risk Score	22.8 ± 2.5	22.8 ± 6.1
HOMA-IR normal (<1.9)	*n* = 1	*n* = 3
HOMA-IR early insulin resistance (1.9–2.9)	*n* = 2	*n* = 3
HOMA-IR insulin resistance (>2.9)	*n* = 1	*n* = 4

Data are presented as mean ± SD. BMI – Body Mass Index, HOMA-IR - Homeostatic Model Assessment of Insulin Resistance. HOMA-IR was calculated from fasted insulin and glucose levels: insulin × glucose/22.5. HOMA-IR data were only available for 13 out of 14 study participants as venous blood sampling could not be completed for one participant. The Diabetes Risk Score was calculated using https://riskscore.diabetes.org.uk/start and is represented as risk score points.

**Table 2:** Habitual dietary intake at the end of the 12-week intervention or control period.

Dietary component	Intervention period	Control period	*p*
Energy (kcal)	1996 ± 601	1810 ± 553	0.030
Fat (% of total energy)	37 ± 16	36 ± 14	0.085
Protein (% of total energy)	17 ± 5	17 ± 4	0.141
Carbohydrates (% of total energy)	45 ± 15	48 ± 16	0.404
Saturated fat (g)	32 ± 16	28 ± 14	0.112
Free sugars (g)	43 ± 33	40 ± 30	0.734
Salt (g)	6 ± 2	5 ± 2	0.005
Fiber (g)	17 ± 7	15 ± 7	0.029
Strawberries	>1/month	<1/week	0.087
Blueberries	<1/week	<1/week	0.632
Grapes	<1/week	<1/week	0.847
Apples	1/week	1/week	0.665

Data are presented as mean ± SD. Statistical analysis was performed using a two-way ANOVA and Tukey post-hoc tests. Based on calculated basal metabolic rate, we identified that four participants likely underreported dietary intake at the end of the control period, and one participant likely underreported dietary intake at the end of the intervention period. Habitual intake data for strawberries, blueberries, grapes and apples were recorded and converted into numeric values for analysis: more than once per week = 0.29 [or 2/7 days], once per week = 0.14 [or 1/7 days], once per month = 0.03 [or 1/31 days], less than once per month = 0.02 [or 0.5/31 days], none = 0. Statistical analysis was performed using a Wilcoxon rank test.

### 3.2. Effect of intervention with bilberry and grape seed extract on glucose and cholesterol markers

**Figure 3:**
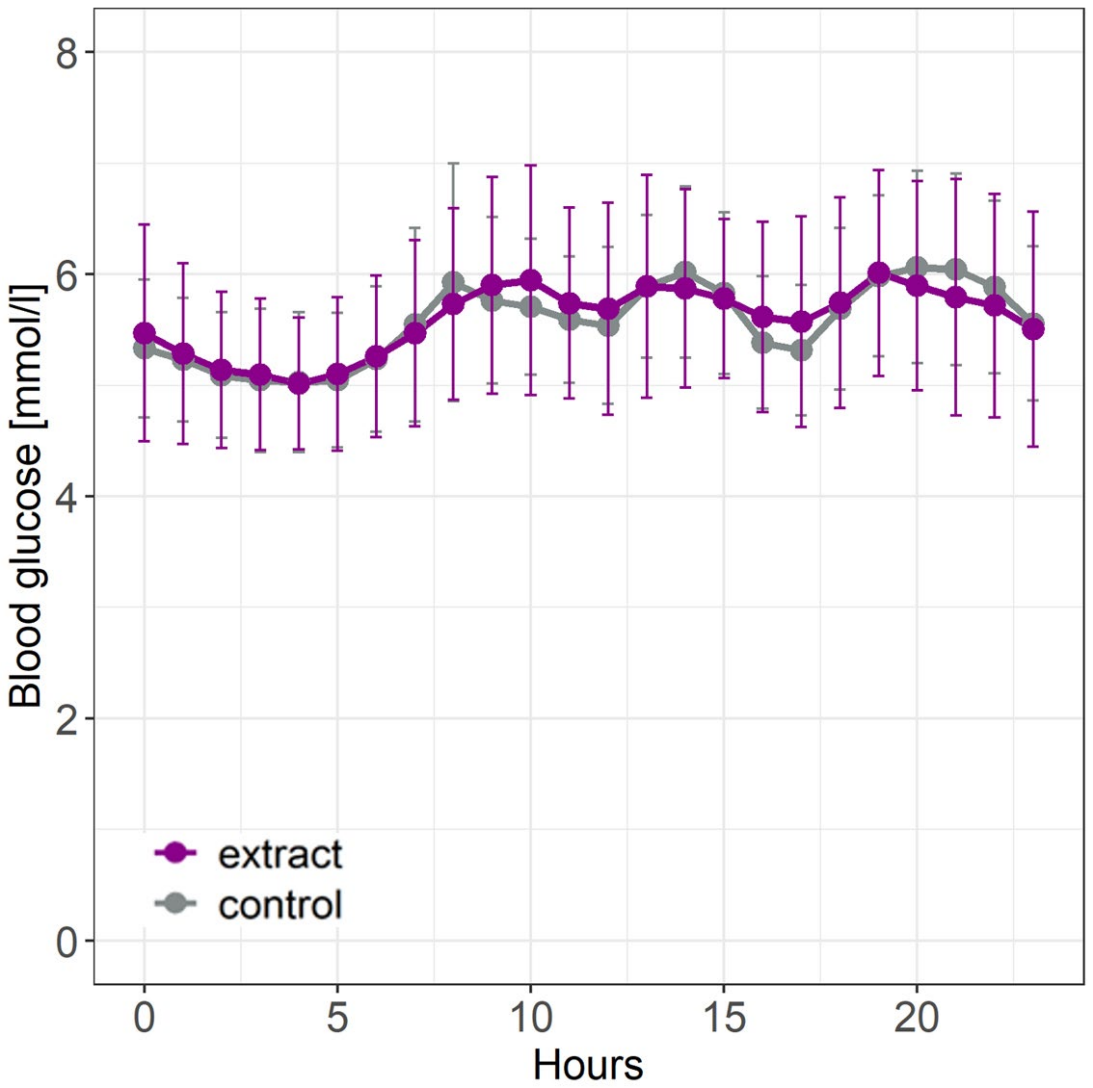
Continuous glucose measurements taken during the last 2 weeks of intervention with bilberry and grape seed extract or control. Data are presented as mean ± SD (*n* = 14 participants). Hourly blood glucose levels were measured using a FreeStyle Libre continuous glucose monitor. Statistical analysis was performed using an ANOVA and Tukey post-hoc test.

Intervention with bilberry and grape seed extract did not affect levels of fasting HbA1c, fasting glucose, 2 hour OGTT results, fasting insulin and glucagon, HOMA-IR, the AGE risk score, total, LDL and HDL cholesterol, compared with control (Table 3). Intervention with bilberry and grape seed extract also did not affect continuous blood glucose measurements, taken over a period of 2 weeks, compared with control (Figure 3). We observed week-to-week variations in HbA1c levels within each participant (Supplementary Figure S2).

**Table 3:** Markers of glucose and cholesterol metabolism after 12-week intervention with bilberry and grape seed extract, compared with placebo extract.

	Baseline	Intervention period	Control period
HbA1c (%)	5.50 ± 0.32	5.42 ± 0.37	5.61 ± 0.40
Fasting blood glucose (mmol/l)	4.89 ± 0.57	4.86 ± 0.40	4.80 ± 0.43
OGTT – 2 hour blood glucose (mmol/l)	6.23 ± 1.64	5.70 ± 1.27	5.49 ± 0.86
Insulin (mU/l)	10.11 ± 3.65	10.41 ± 3.34	10.71 ± 3.63
Glucagon [pg/ml]	571.55 ± 195.25	685.55 ± 284.87	649.49 ± 152.06
HOMA-IR	2.20 ± 0.85	2.27 ± 0.76	2.26 ± 0.76
AGE risk score	--	2.15 ± 0.56	2.16 ± 0.50
Total cholesterol (mmol/l)	5.74 ± 1.47	5.38 ± 1.33	5.55 ± 1.18
LDL cholesterol (mmol/l)	3.53 ± 1.36	3.28 ± 1.36	3.57 ± 0.95
HDL cholesterol (mmol/l)	1.53 ± 0.42	1.49 ± 0.32	1.41 ± 0.43

Data represent measurements taken at baseline, week 12 of the intervention and control periods, and are presented as means ± SD (*n* = 14 participants). Statistical analysis was performed using a two-way ANOVA and Tukey post-hoc tests. HOMA-IR data were only available for 13 out of 14 study participants as venous blood sampling could not be completed for one participant. There are no baseline measurements available for AGE risk scores.

### 3.3. Effect of bilberry and grape seed extract intervention on blood pressure

**Figure 4:**
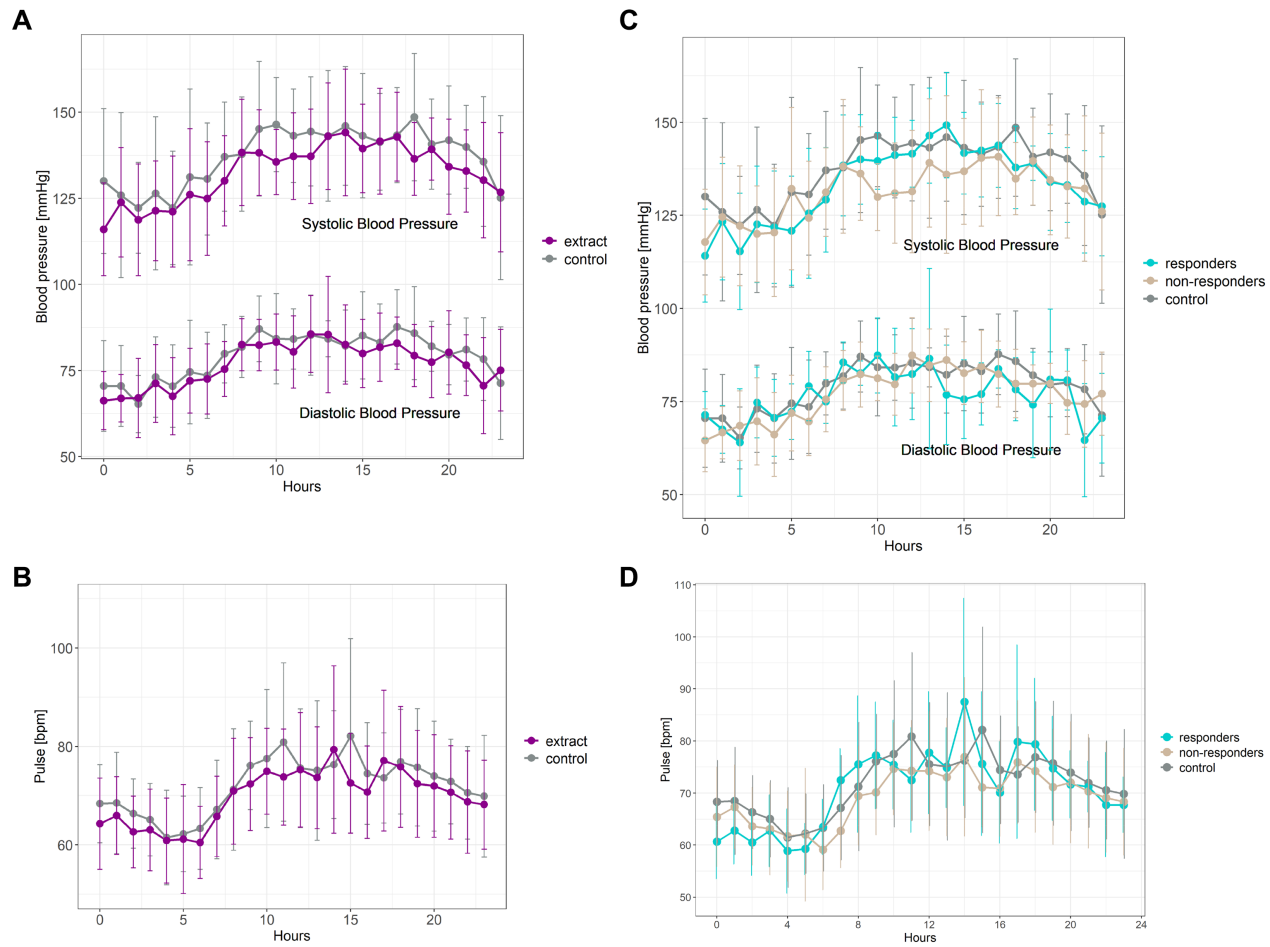
Ambulatory blood pressure **(A)** and pulse measurements **(B)** taken after 8 weeks of intervention with bilberry and grape seed extract (purple) or control (grey). Data are presented as mean ± SD (*n* = 14 participants) as hourly measurements over 24 hours. Statistical analysis was performed using a two-way ANOVA and Tukey post-hoc tests. Systolic blood pressure responders **(C)** (*n* = 8, blue) compared to non-responders (*n* = 6, beige) and control intervention period (*n* = 14, grey). Diastolic blood pressure responders **(C)** (*n* = 5, blue) compared to non-responders (*n* = 9, beige) and control intervention period (*n* = 14, grey). Pulse responders **(D)** (*n* = 4, blue) compared to non-responders (*n* = 10, beige) and control intervention period (*n* = 14, grey).

Intervention with bilberry and grape seed extract significantly decreased 24 hours systolic and diastolic blood pressure, and 24 hours pulse, compared with control intervention (*F*(1,561) = 25.18, p = <0.001; *F*(1,561) = 11.21, *p* = 0.0009, and *F*(1,561) = 13.22, *p* = 0.0003, respectively) (Figures 4A,B). Blood pressure lowering across 24 hours was equivalent to an average decrease in systolic and diastolic blood pressure of 4.8 ± 15.5 mmHg and 2.6 ± 12.1 mmHg, respectively. Eight participants showed a significant (*p* < 0.05) decrease in systolic blood pressure (subsequently identified as blood pressure ‘responders’), five participants showed a significant (*p* < 0.05) decrease in diastolic blood pressure, and four participants showed a significant (*p* < 0.05) decrease in pulse upon intervention with bilberry and grape seed extract (Figures 4C,D; Supplementary Table S1).

### 3.4. Effect of bilberry and grape seed extract intervention on fecal and plasma levels of phenolic metabolites

Intervention with bilberry and grape seed extract did not affect levels of total phenolic metabolite profiles in fecal samples, compared with control, except for levels of protocatechuic acid, of which levels were significantly increased after intervention with bilberry and grape seed extract (*F*(1,24) = 11.66, *p* = 0.002) (Table 4).

**Table 4:** Levels of phenolic metabolites in fecal water and plasma from samples collected in the last week of the intervention and control periods.

	Metabolite	Fecal water			Plasma
		Bilberry and grape seed extract (ng/ml)	Control (ng/ml)	*p*		Baseline	Bilberry and grape seed extract (ng/ml)	Control (ng/ml)	*p*
Benzoic acids	salicylic acid	69.7 ± 60.3	109.8 ± 206.7	NS	15.4 ± 12.6	14.2 ± 10.2	17.0 ± 19.1	NS
	m-hydroxybenzoic acid	405.1 ± 261.6	445.1 ± 209.0	NS	ND	ND	ND	
	p-hydroxybenzoic acid	702.7 ± 1018.4	615.6 ± 682.5	NS	30.0 ± 4.3	31.2 ± 4.8	30.0 ± 6.2	NS
	2,3-dihydroxybenzoic acid	73.8 ± 65.3	63.7 ± 55.2	NS	ND	ND	ND	
	2,5-dihydroxybenzoic acid	698.1 ± 645.2	899.3 ± 659.4	NS	ND	ND	ND	
	2,6-dihydroxybenzoic acid	ND	ND		1.8 ± 3.6	5.5 ± 10.6	4.3 ± 7.4	NS
	protocatechuic acid	443.0 ± 237.2	158.9 ± 171.9	0.002	ND	ND	ND	
	p-anisic acid	4.3 ± 15.5	10.1 ± 36.6	NS	ND	ND	ND	
	vanillic acid	257.3 ± 140.4	168.3 ± 155.9	NS	ND	ND	ND	
	syringic acid	655.8 ± 872.2	279.6 ± 546.4	NS	ND	ND	ND	
Benzaldehydes	p-hydroxybenzaldehyde	113.7 ± 90.1	147.9 ± 166.4	NS	ND	ND	ND	
	protocatachaldehyde	46.5 ± 35.0	37.9 ± 29.7	NS	ND	ND	ND	
	3,4,5-trihydroxybenzaldehyde	83.4 ± 85.2	53.6 ± 120.1	NS	ND	ND	ND	
	vanillin	4.8 ± 6.7	4.1 ± 8.6	NS	ND	ND	ND	
	syringin	42.2 ± 40.7	19.2 ± 37.6	NS	ND	ND	ND	
Cinnamic acids	cinnamic acid	37.5 ± 41.7	23.7 ± 31.8	NS	13.8 ± 21.7	12.0 ± 10.8	15.9 ± 21.1	NS
	p-coumaric acid	124.4 ± 127.3	100.9 ± 133.0	NS	ND	ND	ND	
	caffeic acid	1109.9 ± 2153.4	514.0 ± 982.9	NS	ND	ND	ND	
	ferulic acid	1408.6 ± 2210.2	1101.2 ± 1935.9	NS	ND	ND	ND	
	sinapic acid	38.4 ± 68.4	153.3 ± 395.4	NS	ND	ND	ND	
Phenylpropionic acids	phenylpropionic acid	33255.4 ± 18006.1	30511.8 ± 19741.4	NS	ND	ND	ND	
	2-hydroxyphenylpropionic acid	348.7 ± 301.2	308.8 ± 509.0	NS	ND	ND	ND	
	3-hydroxyphenylpropionic acid	16183.9 ± 27679.0	11545.2 ± 19874.0	NS	ND	ND	ND	
	4-hydroxyphenylpropionic acid	1602.1 ± 2136.5	2802.5 ± 4738.6	NS	ND	ND	ND	
	3,4-dihydroxyphenylpropionic acid	2402.6 ± 1950.9	2606.3 ± 3465.8	NS	ND	ND	ND	
	4-hydroxy-3-methoxyphenylpropionic acid	1808.1 ± 1809.5	1077.0 ± 817.8	NS	2.7 ± 6.1	ND	0.9 ± 2.1	NS
	3-methoxyphenylpropionic acid	37.1 ± 97.8	ND		ND	ND	ND	
Benzenes	1,2-hydroxybenzene	223.8 ± 170.9	131.1 ± 149.3	NS	ND	ND	ND	
Phenylacetic acids	phenylacetic acid	64771.4 ± 28723.7	73625.0 ± 26705.0	NS	644.1 ± 68.0	624.4 ± 56.3	641.0 ± 59.3	NS
	3-hydroxyphenylacetic acid	8639.3 ± 5549.3	7143.9 ± 7164.2	NS	4.9 ± 17.0	6.1 ± 21.1	9.7 ± 33.7	NS
	4-hydroxyphenylacetic acid	8003.6 ± 8684.4	9075.0 ± 12345.0	NS	ND	ND	ND	
	3,4-dihydroxyphenylacetic acid	1464.3 ± 3925.5	1404.9 ± 3814.4	NS	ND	ND	ND	
	4-hydroxy-3-methoxyphenylacetic acid	1869.6 ± 2258.0	1361.4 ± 2204.0	NS	ND	ND	ND	
Phenyllactic acids	4-hydroxyphenyllactic acid	4029.3 ± 3552.8	4111.8 ± 3273.1	NS	52.0 ± 14.5	51.2 ± 8.3	47.9 ± 20.1	NS
Phenolics - other	chlorogenic acid	82.2 ± 227.5	13.0 ± 46.7	NS	ND	ND	ND	
	hydroxyhippuric acid	8.7 ± 21.6	16.2 ± 26.2	NS	ND	ND	1.5 ± 5.3	NS
	tyrosol	307.4 ± 90.2	300.1 ± 114.1	NS	ND	ND	ND	
	hydroxytyrosol	54.4 ± 50.3	45.5 ± 73.6	NS	ND	ND	ND	
Phenolic - dimers	resveratrol	1.1 ± 2.8	ND		ND	ND	ND	
Indoles	indole-3-acetic acid	2630.6 ± 2304.5	2916.1 ± 2814.8	NS	178.4 ± 98.3	196.6 ± 181.7	194.6 ± 140.6	NS
	indole-3-propionic acid	1473.1 ± 1114.7	1264.0 ± 941.3	NS	42.5 ± 32.0	59.0 ± 30.9	72.3 ± 39.8	NS
	indole-3-carboxylic acid	31.2 ± 20.0	45.0 ± 37.1	NS	1.3 ± 1.5	1.9 ± 2.0	1.9 ± 1.8	NS
Flavanoids/Coumarins	catechin	55.3 ± 107.2	48.3 ± 97.1	NS	ND	ND	ND	
	epicatechin	49.6 ± 74.4	46.0 ± 77.1	NS	ND	ND	ND	
	epigallocatechin	4.9 ± 17.6	6.6 ± 16.3	NS	ND	ND	ND	
	isoliquiritigenin	3.6 ± 50.0	3.4 ± 4.8	NS	ND	ND	ND	
	naringenin	3.4 ± 9.4	0.7 ± 2.6	NS	ND	ND	ND	
	naringin	1.1 ± 3.8	ND		ND	ND	ND	
	hesperitin	22.0 ± 50.7	5.1 ± 8.6	NS	ND	ND	ND		
	kaempferol	4.4 ± 8.6	5.0 ± 10.6	NS	ND	ND	ND	
	morin	22.3 ± 59.7	3.2 ± 8.0	NS	ND	ND	ND	
	quercetin	26.3 ± 24.1	23.8 ± 22.2	NS	ND	ND	ND	
	genistein	9.9 ± 12.3	13.5 ± 28.7	NS	ND	ND	ND	
	hesperidin	1.2 ± 3.1	0.7 ± 2.5	NS	ND	ND	ND	
	quercitrin	1.2 ± 4.4	ND		ND	ND	ND	
	biochanin A	7.2 ± 12.4	6.8 ± 12.1	NS	ND	ND	ND	
	daidzein	8.7 ± 13.5	9.2 ± 14.2	NS	ND	ND	ND	
	luteolin	1.0 ± 3.5	2.4 ± 6.1	NS	ND	ND	ND	
	fisetin	45.9 ± 40.2	38.6 ± 32.1	NS	ND	ND	ND	
	formononetin	0.7 ± 2.4	0.6 ± 2.2	NS	ND	ND	ND	
	apigenin	14.3 ± 10.0	17.2 ± 25.2	NS	ND	ND	ND	

Data are presented as mean ± SD (*n* = 14 participants). Fecal waters were prepared by ultracentrifugation; these fecal water samples were mixed with an internal standard (4:1) and phenolic metabolites were detected via targeted analysis using LC–MS/MS. Statistical analysis was performed using a two-way ANOVA and Tukey post-hoc test. ND, non-detectable; NS, non-significant.

Only a limited number of phenolic metabolites (*n* = 12) were detected in plasma. Intervention with bilberry and grape seed extract did not affect levels of plasma phenolic metabolite concentrations, compared with control (Table 4).

### 3.5. Association between individual blood pressure response, baseline gut microbiota composition, and levels of phenolic metabolites

**Figure 5:**
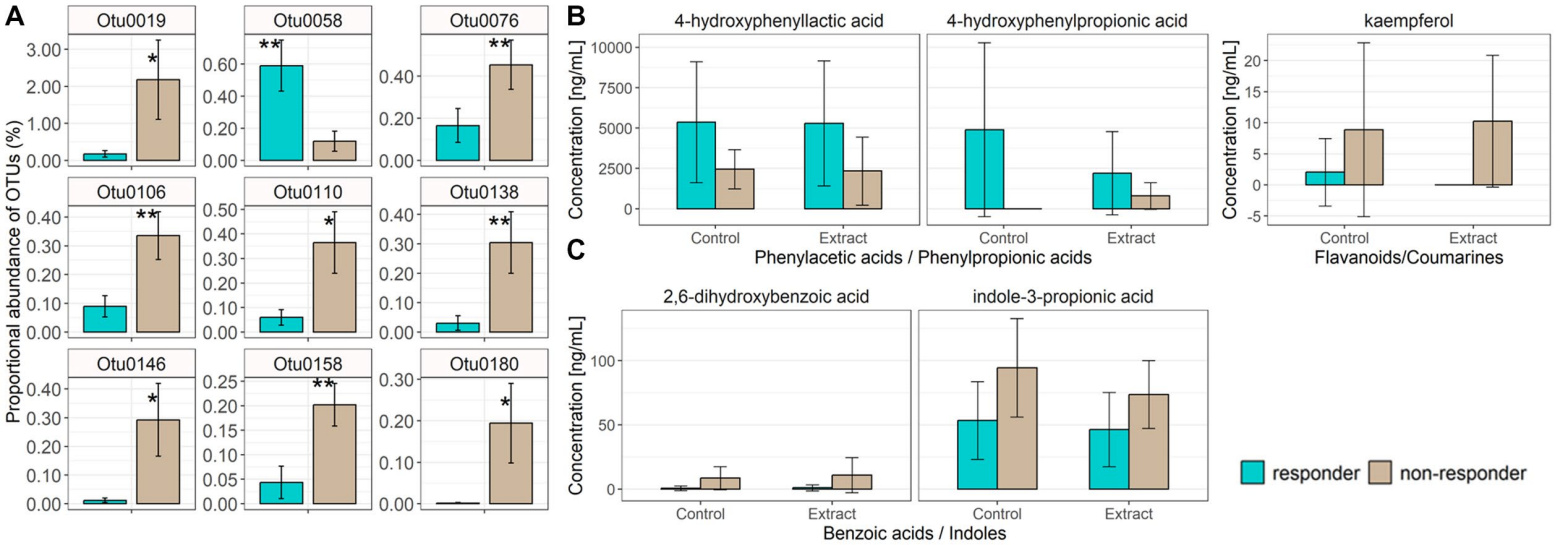
Individual bacterial OTUs that were significantly increased in proportional abundance in either blood pressure responders (mean ± SD, *n* = 8) or non-responders (mean ± SD, *n* = 6), as assessed *via* LEfSe and Metastats **(A)**. Data are represented as percentage of total microbiota sequence reads per sample, mean ± SD. * value of *p* <0.05, ** value of *p* <0.01 obtained from the Metastats calculation. Taxonomic classifications for each OTU are provided in Table 5. Fecal water phenolic metabolites (4-hydroxyphenyllactic acid, 4-hydroxyphenylpropionic acid) were significantly increased in blood pressure responders **(B)** irrespective of control or bilberry and grape seed extract treatment, while kaempferol was significantly lower in blood pressure responders irrespective of control or bilberry and grape seed treatment **(B)**. Blood phenolic metabolites, benzoic acid and indole, were significantly decreased in blood pressure responders irrespective of control or bilberry and grape seed extract treatment **(C)**.

There was no difference in clustering of overall baseline gut microbiota composition between blood pressure responders (i.e., in the eight participants who showed a significant decrease in systolic blood pressure) and non-responders according to parsimony analysis (*p* = 1, and p = 1 respectively), or AMOVA analysis of the Bray-Curtis and Jaccard data (Bray Curtis *p* = 0.302, and Jaccard *p* = 0.222). However, exploratory analysis of individual constituent gut microbial genera revealed that the bacterial genera *Dialister* (LEfSe *p* = 0.033, Metastats p = 0.033), *Collinsella* (LEfSe *p* = 0.028, Metastats *p* = 0.038) and *Coproccocus* (LEfSe *p* = 0.039, Metastats *p* = 0.032) were significantly less proportionally abundant in blood pressure responders compared with non-responders. Furthermore, analysis of individual gut bacterial OTU differences revealed that *Fusicatenibacter-*related OTU0058 was significantly more proportionally abundant in blood pressure responders, while two *Clostridium*-derived OTUs (*Clostridium saudiense*, and *Clostridium disporicum*), a *Coprococcus*, an unclassified *Peptostreptococcaceae*, an unclassified *Firmicutes* and three *Ruminococcaceae* (two unclassified, one *Eubacterium siraeum*) (OTU0019, OTU076, OTU0106, OTU0110, OTU0138, OTU0146, OTU0158, OTU0180) were significantly less proportionally abundant in blood pressure responders (Figure 5A; Table 5). However, no significant differences were observed in gut microbiota genera between blood pressure responders and non-responders after adjusting for multiple comparisons using the Benjamini-Hochberg correction.

**Table 5:** Taxonomic classification of fecal bacteria operational taxonomic units (OTUs) most associated with responder and non-responder status.

OTU	Increased proportional abundance	Closest NCBI BLAST ID	Genus	Phylum
Otu0019	Non-responders	*Clostridium saudiense* (100% similarity)	*Clostridium sensu stricto* (93)	Firmicutes (100)
Otu0058	Responders	*Fusicatenibacter saccharivorans* (96.9% similarity)	*Fusicatenibacter* (100)	Firmicutes (100)
Otu0076	Non-responders	No close cultured match >95% similarity	unclassified *Ruminococcaceae* (100)	Firmicutes (100)
Otu0106	Non-responders	No close cultured match >95% similarity	unclassified *Ruminococcaceae* (74)	Firmicutes (100)
Otu0110	Non-responders	*Coprococcus* sp. L2-50 (100% similarity)	*Coprococcus* (81)	Firmicutes (100)
Otu0138	Non-responders	No close cultured match >95% similarity	unclassified *Firmicutes* (67)	Bacteria unclassified (97)
Otu0146	Non-responders	*Clostridium disporicum* (100% similarity)	*Clostridium sensu stricto* (100)	Firmicutes (100)
Otu0158	Non-responders	*[Eubacterium] siraeum* (100% similarity)	unclassified *Ruminococcaceae* (100)	Firmicutes (100)
Otu0180	Non-responders	Unclassified *Peptostreptococcaceae* sp. (Possible *Romboutsia* sp.? No similarity >98% with cultured species)	unclassified *Peptostreptococcaceae* (100)	Firmicutes (100)

Numbers in brackets in the “Closest BLAST ID” column indicate the percentage sequence similarity to the closest cultured species in the NCBI Nucleotide reference database. Numbers in brackets after the names in the Genus and Phylum column indicate the consistency (out of 100%) of the classifications at each of these taxonomic levels among all sequences within a given OTU using the Ribosomal Database Project reference database.

Levels of 4-hydroxyphenylpropionic acid and 4-hydroxyphenyllactic acid were significantly higher (*p* = 0.026 and *p* = 0.030, respectively), and levels of kaempferol were significantly lower (*p* = 0.024) in fecal waters obtained from blood pressure responders versus non-responders (Figure 5B). Levels of 2,6-hydroxybenzoic acid and indole-3-propionic acid were significantly lower (*p* = 0.004 and *p* = 0.002, respectively) in plasma obtained from blood pressure responders (Supplementary Table S2). Both differences were independent of the intervention (Figure 5C).

## 4. Discussion

A 12-week intervention with a formulated bilberry and grape seed extract did not affect HbA1c levels, a marker of long-term glucose metabolism, nor continuous blood glucose levels, or 2 hour OGTT results, which are both markers of acute glucose metabolism, in participants at risk of developing T2DM. Furthermore, the bilberry and grape seed extract supplement also did not affect total, LDL and HDL cholesterol levels. However, intervention with bilberry and grape seed extract significantly decreased average 24 hours systolic and diastolic blood pressure by 4.8 and 2.6 mmHg, respectively. This reduction in blood pressure is comparable with the efficacy of anti-hypertensive drug treatment, showing an average decrease of 5.9/3.1 mmHg blood pressure across 147 drug intervention studies (51). Such a reduction in blood pressure could reduce the risk for strokes by approximately 10% (52). The bilberry and grape seed extract supplement decreased average 24 hours pulse by 2.3 bpm. Evaluation of individual 24 hours blood pressure responses revealed that eight out of the fourteen participants could be identified as systolic blood pressure responders. Levels of fecal phenolic metabolites 4-hydroxyphenylpropionic acid and 4-hydroxyphenyllactic acid were significantly higher in blood pressure responders compared to non-responders, and kaempferol was significantly lower in blood pressure responders, and this difference was independent of treatment.

To the best of our knowledge, this is the first study where a dietary intervention with both bilberry and grape seed extracts was provided as a single intervention. In a previous review of the literature, we have established that bilberry and grape seed extract products could ameliorate T2DM associated health risks of hyperglycemia, hypercholesterolemia and hypertension (53). Whilst other studies found improvements in HbA1c and cholesterol levels at intervention concentrations of 200–600 mg per day, and with 8 to 12 week intervention periods (14, 53–55), we were not able to replicate these results, even with many of our participants having HbA1c values in the pre-diabetes range (HbA1c ≥5.7%), and cholesterol levels being in the ‘hypercholesterolemia’ range (>5 mmol/l). As we measured both outcomes regularly during the last five weeks of the intervention and control periods, we observed a relatively large week-to-week variation in HbA1c and cholesterol levels, despite both being stable and long-term markers of glucose and cholesterol metabolism, respectively. We did not detect improvements in HbA1c and cholesterol levels upon intervention with bilberry and grape seed extract in our study population, possibly due to the study being underpowered to find significant differences for these outcomes. However, retrospective power calculations based on the PRECISE study data revealed much smaller effect sizes of the bilberry/grape seed extract intervention for HbA1c, and total/HDL/LDL cholesterol outcomes, in our population, compared with those observed in previous studies (31, 34–40). This means we would have required at least 78, 712, 540, or 7,793 participants, respectively, to detect significant changes in these outcomes. This suggests that the effect of bilberry and grape seed extract on glucose and cholesterol metabolism in our participants was negligible.

We did, however, find that intervention with bilberry and grape seed extract significantly lowered blood pressure, as has been observed in other studies in overweight or obese study populations with mild hypertension or hypertension (53, 56–59). Similar reductions in blood pressure were also observed in an intervention study with 300 mg per day of Enovita^®^ grape seed extract, but in male volunteers only (60). A recent Mendelian randomization study identified that systolic blood pressure posed the highest risk for T2DM development among a range of T2DM risk factors (61). Also, studies found that those who develop hypertension or hypercholesterolemia increase their risk for T2DM five-fold (62–64). It has been suggested that an increase in systolic blood pressure levels could be linked to insulin resistance (63), albeit this hypothesis has thus far not been confirmed by Mendelian randomization studies (61, 62). Obesity and insulin resistance are both causal factors for hypertension (63), as insulin resistance and increased insulin levels have been linked to reduced release of nitric oxide, and activation of the renin-angiotensin pathway, causing blood vessel constriction and consequently high blood pressure (63). A recent meta-analysis of 22 studies found that a reduction in systolic blood pressure by 5 mmHg can reduce the risk for T2DM development by 11% (65).

In this study we set out to investigate whether individual differences in gut microbiota and fecal and plasma metabolites could contribute to individual blood pressure response. There was no difference in clustering of overall baseline gut microbiota composition between blood pressure responders and non-responders according to the dendrogram and parsimony analysis of 16S rRNA gene amplicon data, and there were no significantly different taxa after correcting for multiple comparisons. However, differences in fecal bacterial activity between blood pressure responders and non-responders could impact on the metabolism of phenolic compounds in the colon and could therefore be a factor in explaining the higher levels of the fecal metabolites 4-hydroxyphenylpropionic acid and 4-hydroxyphenyllactic acid, and the lower levels of the fecal metabolite kaempferol, in blood pressure responders compared with non-responders. As these findings were independent of the treatment period, this may indicate distinct differences in flavanol metabolism in responders versus non-responders. Increased abundance of *Fusicatenibacter* has been found previously in an *in vitro* fermentation experiment with mango pulp and peel products (66), however it has thus far not been associated with phenolic metabolite digestion in human interventions. *In vitro* fermentation studies of grape seed products confirm the formation of phenylpropionic acids, phenylacetic acids, benzoic acids and also valeric acids and valerolactones, while the concentrations of catechin, epicatechin and polymeric forms thereof decreased in a fecal fermentation over 48 hours (50, 67). This contrasts with our *in vivo* findings as we found levels of catechins and epicatechins in the fecal water of some of our participants, indicating that these volunteers did not fully metabolize these compounds into smaller phenolic acids (Table 4), and indicates care must be taken when comparing results from *in vitro* fermentation and *in-vivo* intervention studies.

While human intervention studies with grape seed or bilberry extract products have not identified what particular fecal bacterial strains could be involved in the metabolism of the phenolic components, other human interventions studies with blueberry powder (25 g/day for six weeks), and with red wine (250–272 ml/day for 20–30 days), identified an increase in fecal *Bifidobacterium* and *Lactobacillus* species after the interventions (68). In an *in vitro* fermentation study with catechin and epicatechin, using individual fecal bacterial strains, *Eggerthella lenta* was identified to be capable of C-ring fission cleavage on catechin and epicatechin, yielding 1-(3,4-dihydroxy- phenyl)-3-(2,4,6-trihydroxyphenyl)propan-2-ol, and *Flavonifractor plautii* was found to convert this initial metabolite of (epi-)catechin further to valerolactone and valeric acid (69). However, the fecal bacterial strains involved in the bioconversion of valeric acids and valerolactones to phenylpropionic, phenylacetic and benzoic acids are not yet known.

*In vitro* and animal studies have reported blood pressure lowering effects of phenolic metabolites *via* vasodilation, by mediating nitric oxide response, by reducing NADPH-dependent oxidative stress, and by inhibiting angiotensin converting enzyme in the renin-angiotensin aldosterone system (70). For example, an *in vitro* model on excised rat aorta identified a strong vasodilatory effect of 3-hydroxyphenylpropionic acid (71). Phenylpropionic acid and phenyllactic acid detected in the fecal waters of the blood pressure responders could be derived from microbial fermentation of anthocyanins or (epi-)catechins in the colon (11, 12). Anthocyanins are metabolized by gut microbial enzymes into caffeic acid and then further metabolized into hydroxyphenylpropionic acids, while catechin/epicatechin polymers are metabolized into monomers and further metabolized into valerolactones, valeric acids and to hydroxyphenylpropionic acids, respectively (11, 12, 69). However, these phenolic acids could also have been formed through the metabolism of unknown precursor metabolites. In the literature there are still contradicting theories about the origin of phenyllactic acids, as phenyllactic acids in fecal waters were weakly correlated with dietary carbohydrates, sugar and starch (72), or phenyllactic acids were proposed to be a product of aromatic amino acid metabolism by the fecal microbiota (30), or they could be fecal metabolism products of dimeric and complex ferulic acids (73).

Main limitations of the PRECISE study were the small number of participants, the unequal ratio of females and males, and the fact that this study was not powered for assessing impacts on blood pressure as this was not a primary outcome. Baseline ambulatory blood pressure was not incorporated into the study design, which limited the statistical approaches we could apply for responder/non-responder analysis. A further potential limitation was that we evaluated the fecal bacterial composition only at baseline, as we did not anticipate significant changes in the gut microbiota population after intervention with bilberry and grape seed extract. However, other studies have reported changes in bacterial composition after short-term dietary changes in animal-based or plant-based foods (74), or after taking epigallocatechin-3-gallate and resveratrol supplements (19). Another limitation was that blood pressure measurements were not taken in the same week as the fecal water samples were obtained, which has to be taken into consideration when assessing the modulation of the blood pressure response by fecal phenolic metabolites.

In conclusion, we found that bilberry and grape seed extract intervention significantly lowered blood pressure, and individual responsiveness in blood pressure was associated with levels of a set of fecal phenolic metabolites that are end-products of (epi-)catechin and anthocyanin metabolism (4-hydroxyphenylpropionic acid, 4-hydroxyphenyllactic acid), blood phenolic metabolites (2,6-dihydroxybenzoic acid, indole-3-propionic acid) and possibly also with the proportional abundance of specific fecal bacteria. This study is the first longer-term study to investigate how individual fecal microbiota and phenolic metabolite profiles might affect the efficacy of a bilberry and grape seed extract to modulate cardiometabolic risk factors in a population, and in individuals at risk of T2DM. The findings of this study could help to improve the design of future human intervention studies to identify individuals who would most likely be responders to dietary interventions with plant bioactives, or indeed improve the response to intervention by targeted supplementation of prebiotics and phenolic metabolites.

## Data availability statement

The 16S rRNA gene-based fecal microbiota dataset has been deposited in the European Nucleotide Archive (ENA) under project accession number PRJEB59712, https://www.ebi.ac.uk/ena/browser/view/PRJEB59712.

## Ethics statement

The studies involving human participants were reviewed and approved by Rowett Institute Ethics Committee (2018/ROW_PRECI/1). The patients/participants provided their written informed consent to participate in this study.

## Author contributions

TG, BR, AW, WR, NH, and XZ designed the research. TG conducted the research. TG and GH analyzed the data. TG and BR wrote the manuscript. All authors have read, contributed to the article, and approved the final version of the manuscript.

## Funding

By-Health Institute of Nutrition & Health, China, provided funding for this study. The sponsor had no role in the data collection, analysis and interpretation of data, and there were no restrictions regarding the submission of the report for publication. The laboratories of BR, AW, WR, and NH are funded by the Scottish Government Rural and Environment Science and Analytical Services Division (RESAS).

## Conflict of interest

XZ is an employee of the company By-health Co., Ltd., and BR is a member of By-Health’s academic advisory board.

The remaining authors declare that the research was conducted in the absence of any commercial or financial relationships that could be construed as a potential conflict of interest.

## Publisher’s note

All claims expressed in this article are solely those of the authors and do not necessarily represent those of their affiliated organizations, or those of the publisher, the editors and the reviewers. Any product that may be evaluated in this article, or claim that may be made by its manufacturer, is not guaranteed or endorsed by the publisher.

## Footnotes

^1^https://www.calculator.net/bmr-calculator.html
