# Supplementary material for: A grape seed and bilberry extract reduces blood pressure in individuals at risk of developing type 2 diabetes: the PRECISE study, a double-blind placebo-controlled cross-over intervention study
Source: Front Nutr. 2023 Jun 7;10:1139880. doi: 10.3389/fnut.2023.1139880 (PMC10283353; doi:10.3389/fnut.2023.1139880)
Supplement: Supplementary file 1 [file Data_Sheet_1.docx]

Supplementary Material

A grape seed and bilberry extract reduces blood pressure in individuals at risk of developing Type 2 Diabetes: the PRECISE study, a double-blind placebo-controlled cross-over intervention study

**Teresa Grohmann, Alan W. Walker, Wendy R. Russell, Nigel Hoggard, Xuguang Zhang, Graham Horgan, Baukje de Roos**

**Correspondence:** Baukje de Roos, Rowett Institute, University of Aberdeen, Foresterhill, Aberdeen AB25 2ZD, United Kingdom. E-mail: b.deroos@abdn.ac.uk

# Data availability: Data generated and analyzed for this study can be requested from the corresponding author. Microbiota sequence data will be uploaded to the European Nucleotide Archive prior to publication.

# Supplementary Figures and Tables


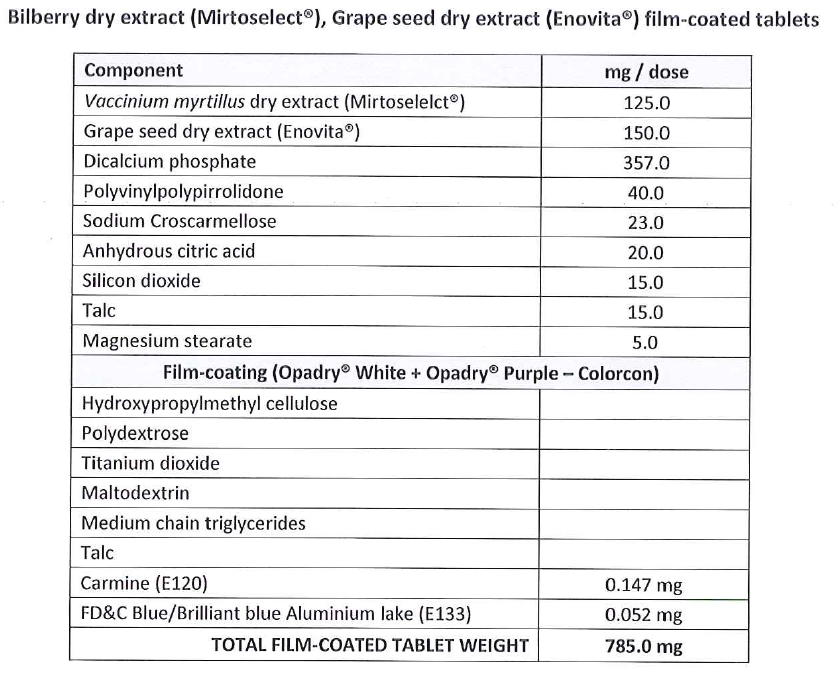

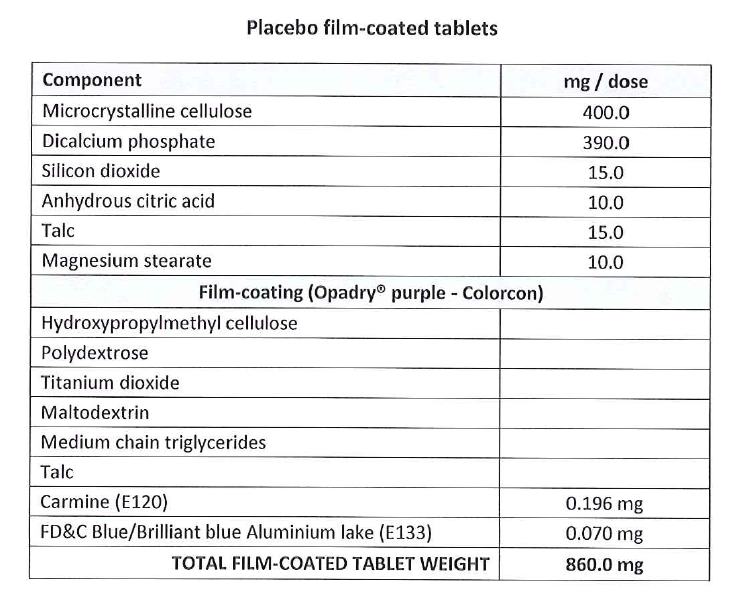


**Supplementary Figure 1**. Composition of intervention and control capsules, as provided by Indena, Italy.


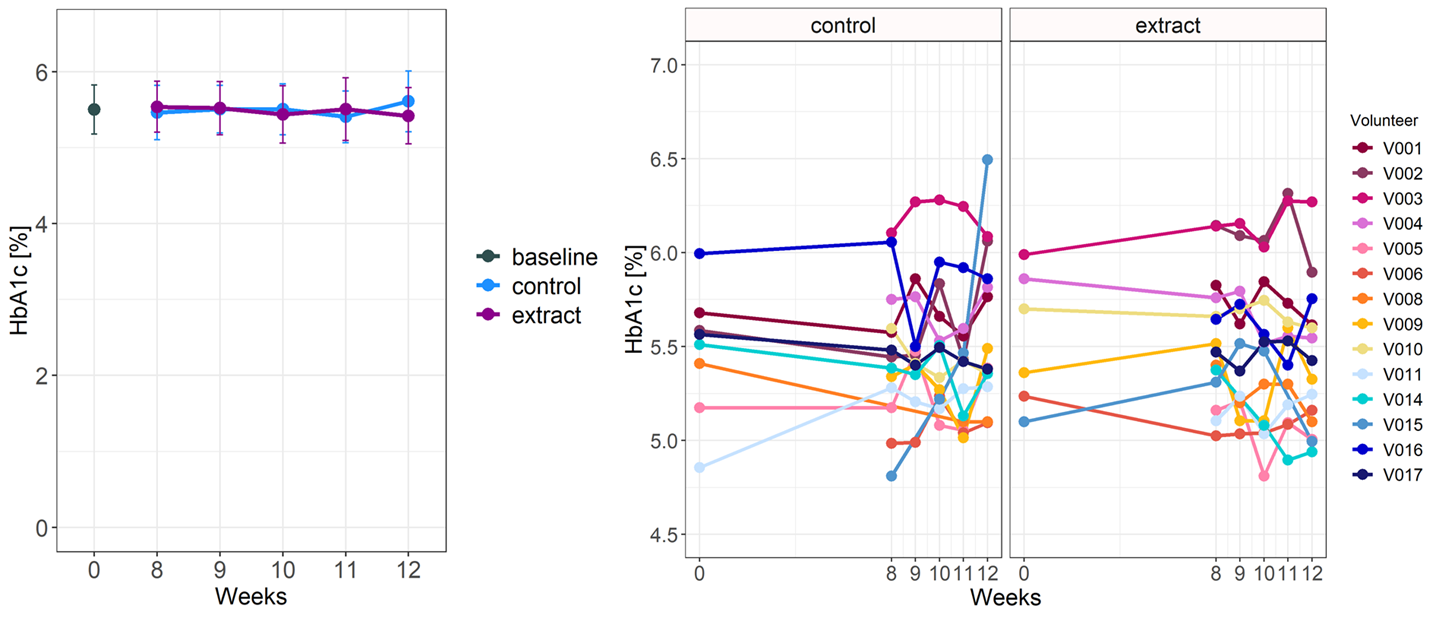


Supplementary Figure 2. Individual levels of HbA1c measured at baseline and weekly after week 8, 9, 10, 11 and 12 of both the intervention and control periods. Data points are means of duplicate analysis.

**Supplementary Table 1**: Identification of systolic and diastolic blood pressure, and pulse, responders and non-responders to intervention with grape seed and bilberry extract.

| **Participant** | **P_SBP_** | **Treatment** | **P_DBP_** | **Treatment** | **P_pulse_** | **Treament** |
| --- | --- | --- | --- | --- | --- | --- |
| V001 | 0.298 |  | 0.171 |  | 0.320 |  |
| V002 | 0.025 | extract | 0.265 |  | 0.233 |  |
| V003 | 0.002 | extract | <0.001 | extract | 0.319 |  |
| V004 | 0.003 | extract | 0.124 |  | >0.001 | extract |
| V005 | 0.030 | control | 0.083 |  | 0.575 |  |
| V006 | 0.123 |  | 0.624 |  | 0.027 | extract |
| V008 | 0.005 | extract | 0.054 | extract | 0.122 |  |
| V009 | 0.104 |  | 0.561 |  | 0.341 |  |
| V010 | <0.001 | extract | <0.001 | extract | 0.040 | extract |
| V011 | 0.001 | control | 0.340 |  | 0.765 |  |
| V014 | 0.006 | extract | 0.045 | extract | 0.874 |  |
| V015 | 0.542 |  | 0.091 |  | 0.330 |  |
| V016 | 0.010 | extract | 0.998 |  | 0.584 |  |
| V017 | <0.001 | extract | 0.013 | extract | <0.001 | extract |

Supplementary Table 2: Plasma levels of phenolic metabolites at baseline, and after extract and control treatment, in blood pressure responders (n=8) and non-responders (n=6).

|  | Blood pressure responders (n=8) | | | Blood pressure non-responders (n=6) | | | P |
| --- | --- | --- | --- | --- | --- | --- | --- |
|  | **baseline** | **control** | **extract** | **baseline** | **control** | **extract** |  |
|  | *Concentration [ng/mL]* | | | | | |  |
| salicylic acid | 16.1 ± 12.6 | 11.2 ± 5.4 | 11.2 ± 6.2 | 14.6 ± 12.6 | 23.6 ± 26.0 | 17.6 ± 12.6 | 0.243 |
| p-hydroxybenzoic acid | 29.5 ± 5.3 | 29.3 ± 6.4 | 30.5 ± 4.7 | 30.7 ± 2.6 | 30.8 ± 5.8 | 32.0 ± 4.8 | 0.444 |
| 2,6-dihydroxybenzoic acid | ND | 0.7 ± 1.8 | 1.0 ± 2.4 | 4.0 ± 4.4 | 8.5 ± 9.0 | 10.9 ± 13.6 | 0.004 |
| cinnamic acid | 18.9 ± 28.6 | 18.1 ± 25.6 | 13.6 ± 13.9 | 7.9 ± 1.5 | 13.3 ± 13.6 | 10.0 ± 4.4 | 0.313 |
| 4-hydroxy-3-methoxyphenylpropionic acid | 3.1 ± 7.7 | 0.9 ± 2.2 | ND | 2.2 ± 3.4 | 0.9 ± 1.9 | ND | 0.809 |
| phenylacetic acid | 651.1 ± 40.0 | 629.8 ± 33.1 | 603.6 ± 49.5 | 635.9 ± 89.6 | 654.2 ± 77.7 | 648.8 ± 53.8 | 0.389 |
| 3-hydroxyphenylacetic acid | 9.1 ± 22.3 | 18.1 ± 44.3 | 11.3 ± 27.6 | ND | ND | ND | 0.136 |
| 4-hydroxyphenyllactic acid | 50.6 ± 18.4 | 48.9 ± 26.3 | 51.0 ± 4.3 | 53.6 ± 7.5 | 46.7 ± 8.2 | 51.6 ± 11.2 | 0.930 |
| hydroxyhippuric acid | ND | ND | ND | ND | 3.3 ± 7.5 | 0 ± 0 | 0.284 |
| indole-3-acrylic acid | 184.8 ± 122.9 | 217.8 ± 182.4 | 226.0 ± 240.0 | 171.0 ± 56.6 | 167.4 ± 51.5 | 162.2 ± 46.1 | 0.396 |
| indole-3-propionic acid | 25.5 ± 23.4 | 53.4 ± 30.2 | 46.4 ± 28.9 | 62.3 ± 29.2 | 94.4 ± 38.3 | 73.6 ± 26.4 | 0.002 |
| indole-3-carboxylic acid | 1.2 ± 1.7 | 1.9 ± 2.0 | 1.8 ± 2.3 | 1.3 ± 1.3 | 2.0 ± 1.5 | 2.1 ± 1.7 | 0.783 |

Data are presented as mean ± SD. Statistical analysis was performed via two-way ANOVA and Tukey post-hoc test.

**
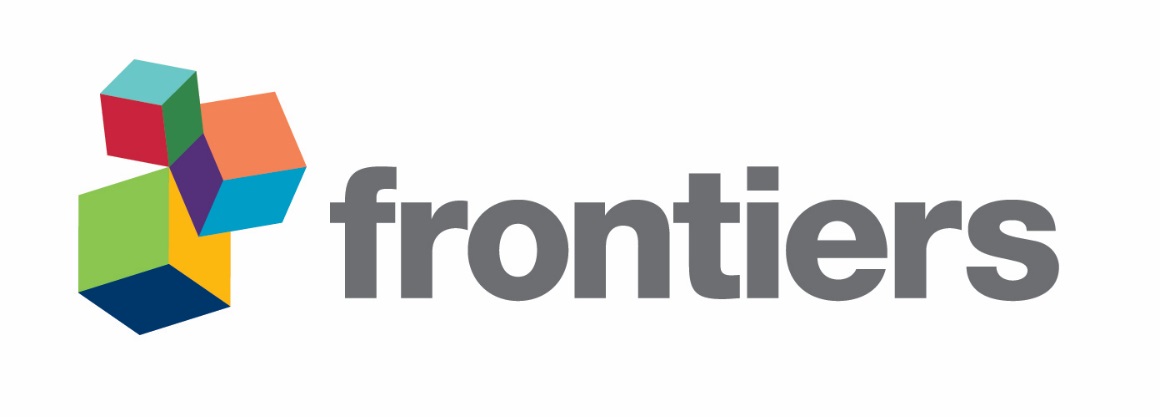
**
